# The Roles of ApoC-III on the Metabolism of Triglyceride-Rich Lipoproteins in Humans

**DOI:** 10.3389/fendo.2020.00474

**Published:** 2020-07-28

**Authors:** Jan Borén, Chris J. Packard, Marja-Riitta Taskinen

**Affiliations:** ^1^Department of Molecular and Clinical Medicine, University of Gothenburg, Gothenburg, Sweden; ^2^Institute of Cardiovascular and Medical Sciences, University of Glasgow, Glasgow, United Kingdom; ^3^Research Programs Unit, Clinical and Molecular Metabolism, University of Helsinki, Helsinki, Finland

**Keywords:** apoC-III, triglycerides, lipoproteins, lipids, cardiovascular disease

## Abstract

Cardiovascular disease (CVD) is the leading cause of death globally. It is well-established based on evidence accrued during the last three decades that high plasma concentrations of cholesterol-rich atherogenic lipoproteins are causatively linked to CVD, and that lowering these reduces atherosclerotic cardiovascular events in humans (1–9). Historically, most attention has been on low-density lipoproteins (LDL) since these are the most abundant atherogenic lipoproteins in the circulation, and thus the main carrier of cholesterol into the artery wall. However, with the rise of obesity and insulin resistance in many populations, there is increasing interest in the role of triglyceride-rich lipoproteins (TRLs) and their metabolic remnants, with accumulating evidence showing they too are causatively linked to CVD. Plasma triglyceride, measured either in the fasting or non-fasting state, is a useful index of the abundance of TRLs and recent research into the biology and genetics of triglyceride heritability has provided new insight into the causal relationship of TRLs with CVD. Of the genetic factors known to influence plasma triglyceride levels variation in *APOC3*- the gene for apolipoprotein (apo) C-III - has emerged as being particularly important as a regulator of triglyceride transport and a novel therapeutic target to reduce dyslipidaemia and CVD risk (10).

## Structure and Regulation of APOC-III

*APOC3* is expressed in hepatocytes and, to a lesser extent in enterocytes (11). It encodes apoC-III, a smaller apolipoprotein of 79 amino acid residues (12). In the circulation, apoC-III is mainly present on TRLs and high density lipoprotein (HDL), and to a lesser extent also on LDL particles (13–16). The distribution of apoC-III between these lipoproteins depends on the metabolic status of individuals, varying between the fasting and postprandial state, and between subjects with normal plasma triglyceride levels and those with hypertriglyceridemia (17–20). Despite the fact that apoC-III was discovered more than 50 years ago (21), we still lack a detailed molecular understanding on how it interacts with lipoprotein particles, enzymes, and cell surface receptors (12, 22–24). However, the two amphipathic helices, and the aromatic tryptophan residues in the carboxyl-terminal half of apoC-III seem to be important for its ability to interact with TRLs (25). Once synthesized, apoC-III can undergo posttranslational modification on threonine-74 resulting in three different glycoforms; unsialylated apoC-III_0_, monosialylated apoC-III_1_ and disialylated apoC-III_2_ (26). The impact of this posttranslational modification has for long been unclear, but recent results indicate that the glycoforms are cleared differently by liver receptors (27).

**Figure 1:**
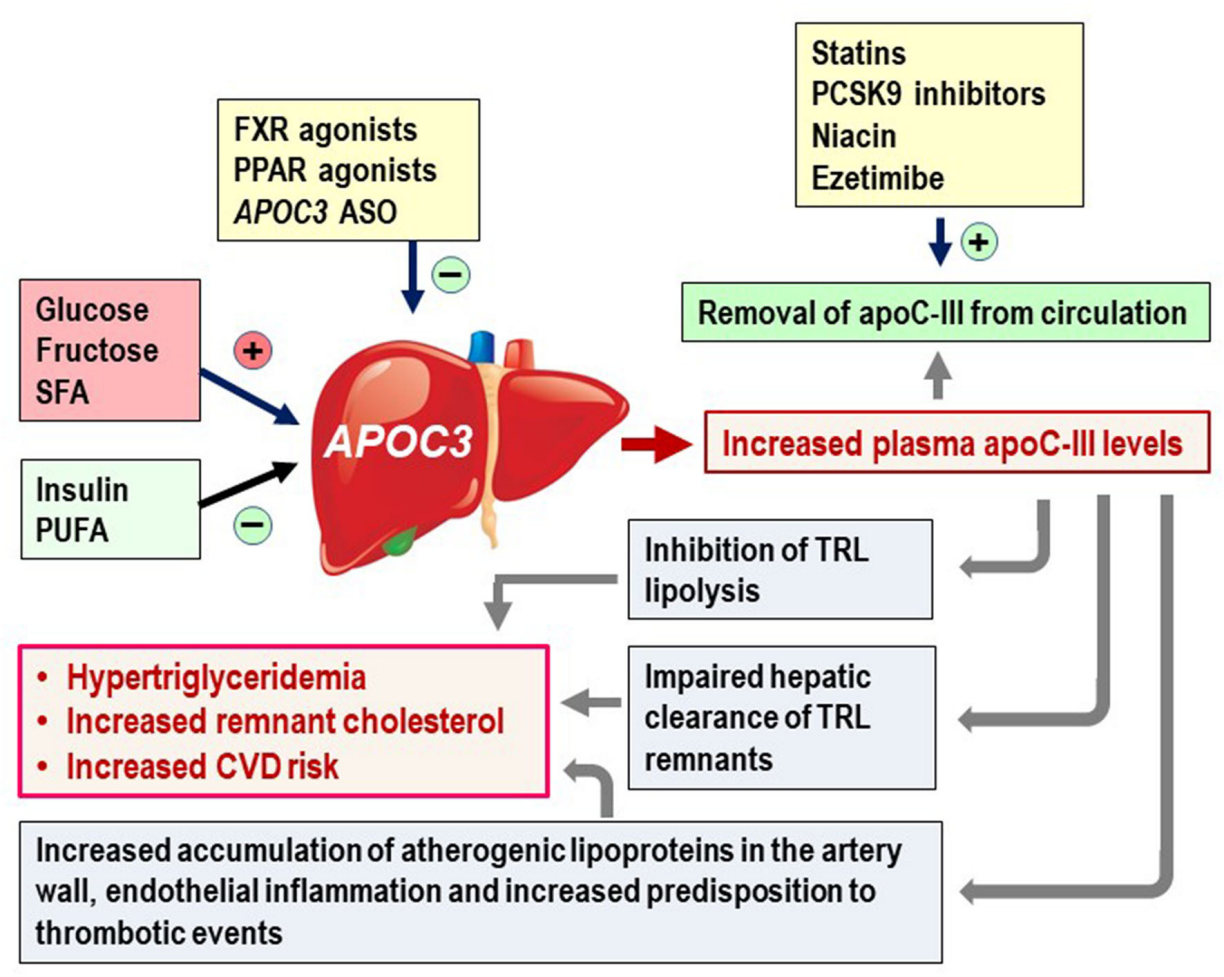
ApoC-III is an important mediator of an atherogenic dyslipidaemia and increased CVD risk. The hepatic *APOC3* expression is induced by carbohydrates (glucose and fructose) and saturated fatty acid (SFA), and reduced by insulin and insulin and polyunsaturated fatty acid (PUFA) (18, 19, 31). Pharmacological intervention by FXR (28, 32, 33) and PPAR (30, 33–36) agonists and *APOC3* antisense oligonucleotides (ASO) reduces hepatic *APOC3* expression (19). Statins (37–39), niacin (40), and ezetimide (40) have been shown to lower plasma apoC-III levels. Increased apoC-III levels induce increase plasma levels of triglycerides and remnant cholesterol, and increased CVD risk. The mechanisms involve impaired lipolysis of TRLs and impaired hepatic clearance of TRL remnants. ApoC-III also facilitates subendothelial accumulation of atherogenic lipoproteins in the artery wall by increasing affinity of atherogenic lipoproteins to artery wall proteoglycans, promoting proinflammatory responses and increasing susceptibility to thrombotic events.

The transcription rate of *APOC3* is decreased by insulin (28, 29), peroxisome proliferator-activated receptor-α (PPARα) (30), and farnesoid X receptor (FXR) (Figure 1) (32). In contrast, glucose stimulates expression of *APOC3* via hepatic nuclear factor-4 **(**HNF4) and carbohydrate-responsive element binding protein (ChREBP) (41). It has been proposed that glucose-mediated regulation of APOC3 expression promotes a shift in the energy source for peripheral tissues from fatty acids released by lipolysis of TRLs to increased utilization of blood glucose (28, 31, 41, 42). *APOC3* expression is therefore upregulated in states of insulin resistance (characterized by insulin resistance and hyperglycemia), and recent results demonstrate that glycaemic control is a major determinant of apoC-III secretion rate *in vivo* (as measured by stable isotope technology) and thus plasma apoC-III levels (43). In these studies it was reported also that apoC-III metabolism is significantly perturbed in subjects with type 2 diabetes; the apoC-III secretion rate was markedly higher than that seen in BMI-matched non-diabetic controls. Improved glycaemic control with the glucagon-like peptide (GLP)−1 analog liraglutide for 16 weeks reduced the apoC-III secretion rate and as a consequence plasma apoC-III levels (43). These findings demonstrate that glucose homeostasis is an important regulator of apoC-III metabolism, and that the secretion rate of apoC-III is an important driver for the elevation of TRLs in subjects with type 2 diabetes (43).

The regulation of hepatic apoC-III expression is now reasonably well-understood, but much less is known of the control of apoC-III synthesis and secretion in the intestine. Intriguingly, overexpression of apoC-III has been shown to decrease intestinal secretion of dietary triglycerides into lymph due to impaired lipid uptake into enterocytes, and impaired esterification capacity to form triglyceride in the mucosa (44). Likewise, intestinal apoC-III overexpression has been reported to result in the secretion of smaller chylomicrons and a reduced triglyceride secretion from the intestine (45).

There is a low concentration of free (i.e., non-lipoprotein associated) apoC-III in the circulation. This form of the protein is chiefly excreted by the kidney (46). It is of note, therefore, that subjects with moderate chronic kidney disease (CKD) which is associated with mild hypertriglyceridemia display increased plasma apoC-III levels due to delayed apoC-III catabolism (47).

## How Does APOC-III Induce Hypertriglyceridemia and Increased Plasma Levels of Atherogenic Remnant Particles?

Human kinetic studies have demonstrated that about 20% of the variation in plasma triglycerides can be explained by increased hepatic production of large triglyceride-rich VLDL_1_ particles, but that impaired removal of TRLs is the main cause explaining about 55% of the variation of plasma triglyceride levels (Table 1) (48). Furthermore, reduced clearance rates of TRLs in turn are closely associated with increased plasma apoC-III levels (48). Thus, apoC-III is a key regulator of triglyceride metabolism (Table 1). Further, metabolic studies in hypertriglyceridemic subjects have shown that the removal of TRL particles from the circulation is impaired if they are enriched in apoC-III (49). As noted above, an increased plasma level of apoC-III in states associated with insulin resistance has been implicated as a key driver of the hypertriglyceridemia commonly found in people with this condition. However, somewhat surprisingly, despite increased plasma apoC-III levels in type 2 diabetic subjects, the concentration of VLDL-apoCIII does not increase in line with that of VLDL-triglyceride. That is, VLDL particles do not seem to be enriched with apoC-III (50, 51), (although it has been reported that there is an increased concentration of LDL particles carrying apoCIII in subjects with type 2 diabetes) (52). This finding requires further investigation and raises the possibility that it is the free form of apoC-III that is the key modulator of plasma triglyceride levels. Interestingly, Kanter et al. recently reported that plasma apoC-III levels predicted future CVD events in type 1 diabetic subjects with normal triglyceride levels. Also, using two mouse models of T1DM, the authors observed that slowly catabolized lipoproteins, enriched in apoC-III and apoE, may be particularly atherogenic (53, 54). Suppressing *APOC3* expression with anti-sense oligonucleotides (ASO) lowered both plasma apoC-III levels and atherosclerosis (54). Thus, apoC-III seems to drive accelerate the CVD risk both in T2DM and T1DM (54).

**Table 1:** Key predictors of plasma triglycerides.

**Key predictors of plasma triglycerides**	
Synthesis pathway	Liver fat (*r* = 0.46, *p* < 0.01)
	Fat mass (*r* = 0.32, *p* < 0.05)
Clearance pathway	Plasma apoC-III concentration (*r* = 0.84, *p* < 0.001)
	Plasma apoC-II concentration (*r* = 0.60, *p* < 0.001)
	Plasma apoE concentration (*r* = 0.60, *p* < 0.001)

*In a stepwise multivariate regression analysis, liver fat content (P < 0.01) and total fat mass (P < 0.05) were identified as independent predictors of VLDL_1_-triglyceride secretion rate (SR) (48). It was also shown that VLDL_1_-triglyceride SR explained 76% of the variation in total plasma triglycerides. The effects of apoC-III on plasma triglycerides is mainly dependent on lipoprotein-lipase independent pathways of triglyceride metabolism (48). Pearson correlations (r–values) between metabolic characteristics, apolipoproteins, and plasma triglycerides. The synthesis explains ≈ 20% of variation in plasma triglycerides, and the clearance pathway ≈ 55% of variation in plasma triglycerides (48)*.

The metabolic and clinical relevance of the three glycoforms of apoC-III has been unclear, but recent studies have shown that the monosialylated apoC-III_1_ correlates stronger with elevated plasma triglyceride levels than the disialylated apoC-III_2_ (55, 56), and that a higher apoC-III_2_/apoC-III_1_ ratio associated with lower triglyceride levels (55). It has also been shown that the relative abundances of apoC-III_0_ and apoC-III_1_, but not apoC-III_2_, are associated with lower triglyceride levels after weight loss or diet intervention (57). In accordance with this concept, apoC-III_2_ inhibits LPL-mediated hydrolysis of TRLs less efficiently than apoCIII_1_ (46), despite having greater affinity for TRLs (58). Interestingly, Kegulian et al. recently reported that the apoC-III glycoforms are differentially cleared by hepatic receptors. Heparan sulfate proteoglycans (HSPGs), in particular syndecan, seem to preferentially clear apoC-III_2_, whereas apoC-III_1_ is preferentially cleared by low-density lipoprotein receptors (LDLR) and LDLR-related protein 1 receptor (LRP1). Interestingly, volanesorsen (a pharmaceutical ASO for *APOC3*) treatment increased the apoC-III_2_/apoC-III_1_ ratio, by increasing the relative abundance of apoC-III_2_ (by 40%) and decreasing that of apoC-III_1_ (by 15%). Thus, the increased apoC-III_2_/apoC-III_1_ ratio seem to reflect faster clearance of apoC-III_1_.

ApoC-III has also been proposed to increase secretion of VLDL in mice overexpressing apoC-III (59–61). However, suppression of *apoC3* expression in mice using an ASO did not influence VLDL secretion (62), and results from kinetic studies in humans are still lacking.

### Inhibition of LPL-Mediated Lipolysis of TRLs

Clearance of plasma triglycerides is directly linked to the lipolysis of TRLs by lipoprotein lipase (LPL) which is attached to the capillary endothelium in adipose tissue, skeletal muscle and the heart (63). ApoC-III is a potent inhibitor of LPL, explaining why increased levels of plasma apoC-III levels correlate with impaired lipolysis of TRLs (Figure 1). The mechanisms involved are not fully elucidated but seem to include weakened binding of TRLs to the capillary endothelium where LPL is present (64), as well as displacement of the LPL activator apoC-II from the surface of the TRLs (15, 65–68).

### Impaired Hepatic Clearance of TRL Remnants

In addition to directly impairing the lipolytic process apoC-III has a wide range of LPL-independent actions on lipid metabolism (19, 60). For example, apoC-III ASOs were shown to greatly reduce serum triglycerides in subjects with familial chylomicronemia syndrome where there is a genetic deficiency of LPL. It appears that apoC-III can inhibit hepatic clearance of remnants by LPL-independent pathways (Figure 1) (69), possibly by interfering with the binding of apoB and apoE to hepatic lipoprotein receptors including HSPG, LDLR and LRPl (31, 70). Recent results indicate that LDLR and LRPl are involved, since apoC-III ASO treatment in LDLR/LRP1 deficient mice did not lower plasma TG levels (65). The principal ligand on the remnant particles is apoE, and by displacing this protein from the lipoprotein particle surface (66), apoC-III effectively impairs the clearance of remnants (71). As apoC-III displaces both apoC-II and apoE from the lipoproteins, it has been proposed that the apoC-III/apoE ratio on remnant particles predicts the hepatic clearance rate of these lipoproteins (66). Interestingly, Ramms et al. recently proposed a model in which apoE determines the metabolic impact of apoC-III on the metabolism of triglycerides by shifting apoC-III's action from supressing hepatic clearance of TRL to inhibition of LPL (72). The model is based on studies showing that suppressing *APOC3* expression in the absence of apoE did not improve clearance of TRLs, yet significantly decreased plasma triglyceride levels *in vivo* (72). This model is supported by previous clinical studies (66) and by studies using genetically modified Apoc3^−/−^Apoe^−/−^ mice (73). Ramms et al. also showed that the triglyceride-lowering effect induced by apoC-III suppression in the absence of apoE, is mainly due to increased LPL activity in white adipose tissue (WAT) (72). Importantly, the study also demonstrated that the efficiency of volanesorsen to lower plasma triglycerides is not dependent on apoE genotype (72). This is important since apoE3 and apoE4 can bind to LDLR and LRP1, whereas apoE2 does not (72).

## Direct Effects of APOC-III on Atherogenesis

Atherogenesis is initiated by subendothelial accumulation of atherogenic lipoproteins. This is mediated by ionic interactions between positively charged domains in apoB100 (74), and negatively charged artery wall proteoglycans (75). ApoC-III facilitates this interaction by increasing the affinity of LDL for the artery wall proteoglycans (Figure 1) (52, 76–80). LDL enriched with apoC-III also displays markedly altered lipid composition, with significantly reduced amount of sphingomyelin, unesterified cholesterol, and ceramides (52). The loss of these lipids, but not of phosphatidylcholine, likely affects the surface fluidity of the lipoprotein particle (81). Thus, the altered lipid composition in apoCIII-enriched LDL may induce conformational changes in apoB100 that are more favorable for proteoglycan binding (52, 82, 83). In line, also apoCIII-enriched HDL display altered lipid composition, with changes in triglycerides, unesterified cholesterol, free cholesterol, phospholipid and apoAI (84).

Following subendothelial retention, LDL are modified by several enzymes, including sphingomyelinases (SMase). This modification promotes both fusion and aggregation of the retained LDL (85, 86), as well as release of proinflammatory metabolites including arachidonic acid (87). The aggregation of LDL may also drive an inflammatory response as aggregated LDL is a potent inducer of macrophage foam cell formation (88). Interestingly, apoC-III acts as a SMase activator. Thus, apoC-III may promote proatherogenic modification of retained LDL in the artery wall, and induce inflammatory responses (86, 89). ApoC-III has also been shown to directly activate adhesion molecules and proinflammatory responses in monocytes and endothelial cells (Figure 1) (90, 91). In addition, apoC-III levels have also been shown to strongly correlate with plasma levels of activated factor VII-anti-thrombin (FVIIa-AT) complex, a biomarker for increased predisposition to thrombotic events (Figure 1); a strong association was found in both sexes, regardless of whether or not there had been a prior CAD event (92). Thus, apoC-III seems to link lipid metabolism and coagulation. Finally, under conditions of islet insulin resistance, local islet production of apoC-III has been identified as a diabetogenic factor involved in impairment of β-cell function. Thus, apoC-III synthesized in the pancreas seems to link insulin resistance and β-cell failure in T2 DM (93).

Capoulade et al. recently reported that apoC-III is present on lipoprotein (a) (Lp(a) particles in the circulation and in the aortic valve leaflets (94). Their results indicate that increased plasma levels of apoCIII-Lp(a) complexes in combination with Lp(a)-OxPL may be used to predict aortic stenosis and aortic valve replacement (94).

## What Have We Learned From Epidemiology and Genetic Studies?

Epidemiological studies have revealed that plasma levels of apoC-III and apoB independently predict coronary heart disease (1–4). ApoC-III levels even predict coronary events independent of LDL cholesterol values (2–4). In diabetic subjects, those with LDL with the highest apoC-III content have a six-fold higher relative risk of new coronary events compared to those with LDL with lowest apoC-III content (3). Furthermore, Olivieri et al. recently reported that high plasma apoC-III levels predict an increased risk of ischemic stroke/transient ischemic attack (TIA) events in cardiovascular patients (95).

Large genetic studies have demonstrated that elevated plasma triglyceride is causally linked to coronary artery disease (CAD) (5–9). For example, both the Exome Sequencing Project (*n* = 1,10,970) and the Copenhagen Study (*n* = 75,725) reported that *APOC3* LOF mutations had about 40% lower plasma triglycerides and about 40% lower CVD risk. These results suggest that 1 mg/dl decrease in plasma apoC-III concentration translates to a 4% decrease in CVD incidence (8).

Genetic studies have also shown that carriers of the *APOC3* null mutation R19X have 50% lower plasma apoC-III levels, 35% lower plasma triglycerides, markedly lower postprandial triglycerides and significantly lower coronary artery calcification (CAC) scores than non-carriers (96, 97). Thus, lifelong deficiency of apoC-III is cardioprotective. Carriers of the R19X null mutation display both lower apoC-III production rate and increased apoC-III clearance rate, leading to increased lipolysis of TRLs (96). As expected, the lower plasma apoC-III levels did not influence direct VLDL clearance (i.e., removal of VLDL particles) (96). Carriers of the *APOC3* null mutation R19X variant are rare [0.08% in Americans (98) and 0.05% in Europeans (99)], but the R19X variant is enriched in the Amish population and in an isolated cohort on the island of Crete (100). Heterozygote LOF mutations in *APOC3* have also been shown to associate with high HDL-cholesterol in addition to low plasma triglycerides (101, 102). The heterozygote *APOC3* LOF mutation Ala43Thr variant has also been associated with impaired lipidation of nascent VLDL particles during their hepatic assembly (103). Thus, some *APOC3* genetic variants may modulate plasma triglyceride levels by mechanisms other than enhanced lipolysis.

Plasma triglyceride levels closely correlate with remnant cholesterol, and genetic studies in apoC-III LOF carriers have made it possible to analyse if remnant cholesterol independently predict ischemic heart disease (IHD) risk (104). Heterozygotes for *APOC3* LOF mutations had 43% lower remnant cholesterol, minor changes in LDL-cholesterol (mean of −4%), and a 13% lower apoB compared to non-carriers (104). Mediation analysis indicated that about half of the lower risk of IHD in LOF carriers was attributable to the difference in remnant cholesterol and only about 2% to the difference in LDL cholesterol. This result adds to the Mendelian randomization studies by Ference et al. showing that reductions in triglyceride levels do not reduce CVD risk unless there was an accompanying reduction in circulating apoB levels, and that the reduction in risk was proportional to the decrement in apoB (105). The 36% lower IHD risk for a 14 mg/dl lower apoB in the Copenhagen studies (104) is in line with the 23% lower risk per 10 mg/dl decrement in plasma apoB seen by Ference et al. (105). What the former investigation seems to indicate is that is does not matter if the apoB difference is in remnant particles or LDL. This is in line with the notion that any apoB-containing lipoproteins able to penetrate into the artery wall are atherogenic.

As the allele frequency of APOC3 LOF mutations is low, very few homozygous carriers have been identified. However, four homozygotes carriers (Arg19Thr) were recently identified in Pakistan (106). In addition, a family with nine children, all homozygous carriers (Arg19Thr) was recently identified (106). As expected, the homozygotes APOC3 LOF carriers had low very plasma apoC-III levels and markedly blunted postprandial triglyceride responses (106).

## Can Diets Modulate Plasma APOC-III Levels?

Genetic studies clearly show that low plasma levels of apoC-III are cardioprotective. So how can we lower apoC-III? The first option is with dietary intervention. As *APOC3* expression is induced by glucose, it's not surprising that the carbohydrate-content of the diet correlates with plasma apoC-III levels (57, 107–110). For example, fructose-enriched diets have been shown to induce several cardiometabolic risk factors including increased apoC-III plasma levels (111–113) and fructose restriction has been shown to lower plasma apoC-III (112, 114). In line, a two-week intervention using an isocaloric low-carbohydrate diet (<30 g carbohydrates/day) induced an almost 50% reduction of plasma apoC-III levels in obese subjects with non-alcoholic fatty liver disease (NAFLD) (115). Interestingly, fructose seems to have particularly adverse effects on apoC-III levels since it has been observed that subjects consuming fructose for 10 weeks had higher plasma apoC-III levels and postprandial TRL-triglycerides than subjects consuming an equivalent amount of glucose (116). Interestingly, Hieronimus and Stanhope have recently proposed that apoC-III might be causal for fructose-induced dyslipidaemia since suppression of *APOCIII* expression in non-human primates prevented fructose-induced dyslipidemia (117).

Fructose induced not only increased expression of *APOC3* (111, 113), but also increased hepatic *de novo* lipogenesis of fatty acids that is an important initiator of NAFLD and overproduction of triglyceride-rich VLDL_1_ particles (43, 61, 118–122). The relative importance of increased liver fat vs. increased secretion of apoC-III for fructose-induced hypertriglyceridemia, remains to clarified. Consumption of saturated fat has been reported to increase plasma apoC-III levels (42, 123), whereas intake of mono- and poly-unsaturated fat associate with reduced plasma apoC-III levels (Figure 1) (123). Also, omega-3 polyunsaturated fatty acids have been reported to decrease plasma apoC-III levels (124, 125). Whether this mechanism is relevant for their triglyceride-lowering effects remains to be clarified (126).

## Pharmacological Interventions for Reducing Plasma APOC-III Levels and Hypertriglyceridemia

Earlier studies have reported that PPARα agonists reduce *APOC3* and plasma apoC-III levels (30, 33, 127). However, the ability of fibrates to reduce *APOC3* expression is highly variable ranging from 10 to 40% (37, 128–131). Even less has been reported on how PPARγ agonists (pioglitazone, rosiglitazone) affect apoC-III metabolism (34, 132). Also, nicotinic acid (niacin) (133) and statin therapy have been shown to reduce hepatic *APOC3* expression through largely unknown mechanisms (38). Meta-analyses have revealed that statins reduce plasma apoC-III levels (134) and Ooi et al. reported that that the statin rosuvastatin both decreased the production rate of apoC-III, and simultaneously increased its catabolism (38). Omega-3 carboxylic acids (OM3-CA) and polyunsaturated fatty acids have also been shown to reduce plasma apoC-III by 20–30% (125, 135, 136). However, compared to the actions of ASOs, these interventions reduce apoC-III levels only to a moderate degree.

Development of novel technologies including ASOs, siRNAs and monoclonal antibodies (137, 138), as well as improved targeting methods (139, 140), including use of N-acetyl galactosamine-conjugated (GalNAc) adducts (i.e., the ligand of the hepatic asialoglycoprotein receptor), have enabled unprecendented fast translation of basic science to clinical intervention (141). For example, volanesorsen (IONIS-APOCIII Rx) represents a second-generation 2′-O-methoxyethyl (2′-MOE) chimeric antisense therapeutic oligonucleotide that efficiently reduce *APOC3* expression (62).

Results from the recent APPROACH trial, a 52-week randomized, double-blind, phase 3 trial of volanesorsen-mediated inhibition of *APOC3* expression in 66 patients with familial chylomicronemia syndrome, showed that volanesorsen induced a 77% decrease in mean triglyceride levels (mean decrease of 19.3 mmol/l), whereas patients receiving placebo had an 18% increase in mean triglyceride levels. Common adverse events were mild thrombocytopenia and injection-site reactions. These results validate earlier studies showing that apoC-III inhibits not only LPL-dependent but also LPL-independent pathway(s) of TRL clearance (69).

Volanesorsen has in an earlier randomized, double-blind phase 2 trial been shown to markedly lower plasma apoC-III and triglycerides levels in adult patients (*n* = 46) with severe or uncontrolled hypertriglyceridemia (from 4.0 to 22.6 mmol/l) (142). The results showed dose-dependent decreases of both plasma apoC-III and triglyceride levels (about 80 and 71% decreases, respectively). Similar results were reported from the COMPASS study which recruited 113 subjects with severe hypertriglyceridemia (5.7 to 14.8 mmol/l) (137). A critical reason for treating severe hypertriglyceridemia is to reduce the risk of acute pancreatitis. It is therefore promising that acute pancreatitis were markedly less in hypertriglyceridemic patients treated with volanesorsen than in the placebo group (143).

Volanesorsen has also been shown to successfully improved diabetic dyslipidaemia by reducing both apoC-III (−88%) and plasma TG (−69%) in 15 overweight or obese subjects with type 2 diabetes (144). Interestingly, the agent not only improved the dyslipidemia, but also improved whole-body insulin sensitivity (by 57%) as compared to placebo. Thus, results from the novel antisense therapeutic approach seem promising, but data from large-scale and cardiovascular outcome clinical trials are still missing.

The safety, tolerability, and efficacy of AKCEA-APOCIII-LRx, a next generation GalNAc ASO that is targeted to the liver where it suppresses hepatic APOC3 expression, was recently tested. Results showed 89% in reduction in apoC-III levels, and 66% reduction in plasma triglycerides (145).

Another novel strategy to lower plasma triglycerides was recently reported by Wolska et al. (146). They developed a dual apoC-II mimetic and apoC-III antagonist (called D6PV) that activates LPL. The peptide was designed by combining biophysical techniques and advanced molecular simulation of apoC-II. D6PV was shown to be more efficient in activating LPL than full-length apoC-III, and was shown to markedly lower plasma triglycerides (>80%) in both apoC-II–deficient mice and hAPOC3-transgenic mice. The peptide reduced plasma apoC-III levels by 80% and apoB levels by 65%. The peptide remains in the circulation for to 50 h in non-human primates, as it binds to HDL particles. Thus, the results are encouraging but the project is still in early development (147).

## Concluding Remarks and Remaining Questions

Interest in apoC-III as a novel intervention target has been driven by epidemiological studies demonstrating that plasma apoC-III levels predict coronary events independent of LDL cholesterol values (2–4), and genetic studies demonstrating that *APOC3* LOF mutations associate with lower plasma triglycerides and about 40% lower CVD risk. Recent studies have shown that glucose is an important regulator of apoC-III metabolism (19, 43), and that increased hepatic secretion of apoC-III is an important driver for the hypertriglyceridemia commonly seen in subjects with impaired glucose homeostasis (43). The lower CVD risk associated with *APOC3* LOF mutations is likely not related to lower plasma triglycerides *per se*, but may depend on lower plasma concentrations of atherogenic remnant particles. Thus, suppression of hepatic *APOC3* expression has become an interesting novel treatment for reducing hypertriglyceridemia and accumulation of atherogenic remnant particles. However, there are some concerns as the treatment has shown less marked response on apoB reduction, than for example suppression of *ANGPTL3* that seems to reduce plasma apoB levels more efficiently (148). Long-term clinical studies will be critical for clarifying the protective potential of *APOC3* ASO. It will also be interesting to see if this treatment has direct effects on hepatic VLDL secretion, and markers of arterial wall inflammation.

## Author Contributions

All authors listed have made a substantial, direct and intellectual contribution to the work, and approved it for publication.

## Conflict of Interest

The authors declare that the research was conducted in the absence of any commercial or financial relationships that could be construed as a potential conflict of interest.
